# Reliable detection of *Bacillus anthracis*, *Francisella tularensis *and *Yersinia pestis *by using multiplex qPCR including internal controls for nucleic acid extraction and amplification

**DOI:** 10.1186/1471-2180-10-314

**Published:** 2010-12-08

**Authors:** Ingmar Janse, Raditijo A Hamidjaja, Jasper M Bok, Bart J van Rotterdam

**Affiliations:** 1 National Institute for Public Health and the Environment, Laboratory for Zoonoses and Environmental Microbiology, Anthonie van Leeuwenhoeklaan 9, 3721 MA Bilthoven, The Netherlands

## Abstract

**Background:**

Several pathogens could seriously affect public health if not recognized timely. To reduce the impact of such highly pathogenic micro-organisms, rapid and accurate diagnostic tools are needed for their detection in various samples, including environmental samples.

**Results:**

Multiplex real-time PCRs were designed for rapid and reliable detection of three major pathogens that have the potential to cause high morbidity and mortality in humans: *B. anthracis*, *F. tularensis *and *Y. pestis*. The developed assays detect three pathogen-specific targets, including at least one chromosomal target, and one target from *B. thuringiensis *which is used as an internal control for nucleic acid extraction from refractory spores as well as successful DNA amplification. Validation of the PCRs showed a high analytical sensitivity, specificity and coverage of diverse pathogen strains.

**Conclusions:**

The multiplex qPCR assays that were developed allow the rapid detection of 3 pathogen-specific targets simultaneously, without compromising sensitivity. The application of *B. thuringiensis *spores as internal controls further reduces false negative results. This ensures highly reliable detection, while template consumption and laboratory effort are kept at a minimum

## Background

A group of diverse pathogens has the potential to cause high morbidity and mortality in humans -especially if carried by aerosols- even though they do not pose a major threat to public health under normal circumstances. The most menacing bacterial pathogens of this group are *Bacillus anthracis*, *Francisella tularensis *and *Yersinia pestis*, and these organisms are listed as category A biothreat agents (classification of the CDC, USA, http://www.bt.cdc.gov/agent/agentlist-category.asp) because of the potential danger of their deliberate release. Exposure to aerosolized *B*. *anthracis *spores and *F*. *tularensis *can lead to inhalational anthrax and tularemia. *Y. pestis *may cause pneumonic plague, which, unlike the other two diseases, may also spread from person to person.

To reduce the public health impact of such highly pathogenic micro-organisms, rapid and accurate diagnostic tools for their detection are needed. Timely recognition of disease agents will enable appropriate treatment of exposed individuals which will be critical to their survival, and the spread of disease can be reduced by taking appropriate public health measures. Classical identification involves culturing suspect pathogens, but although culturing can be very sensitive, these methods are time consuming, not very specific, involve extensive biosafety measures and some organisms simply resist cultivation. Real-time qPCR methods for the detection of pathogens can be equally or more sensitive, and can also provide higher speed and specificity. Also, molecular methods require only preparatory handling of samples under biosafety conditions and can be easily scaled-up, which is important for speeding up investigations and control of disease progression in outbreak situations. Despite these manifold advantages, detection of DNA does not yield information about the presence of viable organisms.

Multiplexing qPCR detection offers several advantages, including reduction of sample volume and handling time (reducing the analysis time, cost and opportunities for lab contamination). Also, false-negative results can be reduced through co-amplification of internal controls in each sample, and using multiple redundant genetic markers for each organism reduces the chance that strain variants are missed. Amplification of multiple signature sequences per organism will also reduce false-positive results in complex samples. False positives can be an issue if detection relies on single targets when analyzing environmental samples, due to the presence of homologous sequences in related organisms or unknown sources [1,2]. Therefore, it is essential to validate the qPCR using multiple strains, including of closely related organisms.

The selection of suitable signature sequences is an essential requirement for reliable PCR assays. The suitability of signature sequences may be based on their function, e.g. detection of virulence factors supplies important information. But also the stability of their association with the pathogen is of importance. For instance, virulent *B*. *anthracis *can be recognized by its virulence plasmids pXO1 and pXO2 [3] which contain genes that confer toxin production and capsule synthesis activities, respectively. However, there are also chromosomally encoded factors that are important for the full virulence of *B*. *anthracis *[4]. Also, recent studies have shown the occurrence of a plasmid homologous to pXO1 in a pathogenic *B*. *cereus *strain [5] as well as genes homologous to genes on pXO2 in environmental *Bacillus *isolates [2]. This underscores the importance of inclusion of a chromosomal signature for *B. anthracis *in addition to the detection of plasmid genes. Similarly, virulent *Y. pestis *possesses 3 plasmids involved in virulence, but these plasmids are not stable and pathogenic *Y. pestis *lacking any of these plasmids exists [6].

Several reports have described real-time PCR assays for the detection of *B. anthracis *[7-10], *Y. pestis *[6,11,12] and *F. tularensis *[13-15]. Some assays were designed in multiplex format, but only few of these included internal controls for DNA amplification [10,16] and none included an internal control for successful DNA extraction. Here, we report the highly reliable and sensitive detection of these three pathogens that we achieved by developing multiplex qPCRs for 3 organism-specific markers and 1 internal control. By using a *B. thuringiensis *gene as internal control, it is possible to use the highly refractory spores of this near relative of *B. anthracis *as a control for both DNA extraction and qPCR amplification. The assays were extensively validated and were used on different real-time PCR platforms. The multiplex qPCRs are being applied in screening protocols and our setup allows straightforward expansion of the detection capabilities by inclusion of additional pathogens.

## Results

### Design of multiplex hydrolysis probe assays

A selection of signature sequences for the specific detection and partial characterization of *B*. *anthracis*, *F*. *tularensis *and *Y. pestis *was based on previous reports [4-6,8,11-14,17], and sequence data accessible via public databases (NCBI/EMBL). Additional sequences were obtained from *sspE *genes from a number of strains from the *Bacillus cereus *group in our culture collection and from the *cry1 *gene from *B*. *thuringiensis *strain ATCC 29730. Based on signature sequence alignments, regions were identified that were shared by all strain variants and sufficiently different from homologous sequences to design selective oligonucleotides for multiplex real-time qPCR assays (see Table 1).

**Table 1:** Primers and probes for multiplex qPCR

*Organism*	*Target*	*Oligo function*	*Oligo name*	*Sequence 5'-3'^a^*
*B. anthracis*	*sspE*	Forward primer	spEpri_f	CGACTGAAACAAATGTACAAGCAGTA
		Reverse primer	spEpri_r	CGTCTGTTTCAGTTGCAAATTCTG
		Probe	Tqpro_spE	**FAM**-TGCTAGCATTCAAAGCACAAATGCTAGTT-**BHQ1**
	
	*cya*	Forward primer	cyapri_f	AGGTAGATTTATAGAAAAAAACATTACGGG
		Reverse primer	cyapri_r	GCTGACGTAGGGATGGTATT
		Probe	Tqpro_cya	**JOE-**CCACTCAATATAAGCTTTATTACCAGGAGC**-BHQ1**
	
	*capB*	Forward primer	caBpri2_f	AGCAAATGTTGGAGTGATTGTAAATG
		Reverse primer	caBpri2_r	AAAGTAATCCAAGTATTCACTTTCAATAG
		Probe	Tqpro_caB	**CFR590-**AGGTCCCATAACATCCATATGATCTTCTAA**-BHQ2**

*F. tularensis*	*fopA*	Forward primer	foApri_f	GCGCTTTGACTAACAAGGACA
		Reverse primer	foApri_r	CCAGCACCTGATGGAGAGTT
		Probe	Tqpro_foA	**FAM-**TGGCCAGTGGTACTTAGGTGTAGATGCTA**-BHQ1**
	
	IS*Ftu2*	Forward primer	isfpri2_f	CAAGCAATTGGTAGATCAGTTGG
		Reverse primer	isfpri2_r	GACAACAATATTTCTATTGGATTACCTAAA
		Probe	Tqpro_isf	**JOE-**ACCACTAAAATCCATGCTATGACTGATG**-BHQ1**
	
	*pdpD*	Forward primer	pdDpri_f	TCAATGGCTCAGAGACATCAATTAAAAGAA
		Reverse primer	pdDpri_r	CACAGCTCCAAGAGTACTATTTCC
		Probe	Tqpro_pdD	**CFR590-**ACCAAATCAAAATCCTGCTGAGCAGA**-BHQ2**

*Y. pestis*	*ypo393*	Forward primer	yp93pri_f	AGATAGTGTGACTGGTCTTGTTTCA
		Reverse primer	yp93pri_r	AGATGCAGATTGTATTGTAAACAATGAC
		Probe	Tqpro_yp93	**FAM-**ACTTCCTGATATATTGGAAATCTTCTTCTC**-BHQ1**
	
	*caf1*	Forward primer	cafpri_f	CCAGCCCGCATCACT
		Reverse primer	cafpri_r	ATCTGTAAAGTTAACAGATGTGCTAGT
		Probe	Tqpro_caf	**JOE-**AGCGTACCAACAAGTAATTCTGTATCGATG**-BHQ1**
	
	*pla*	Forward primer	plapri_f	ATGAGAGATCTTACTTTCCGTGAGAA
		Reverse primer	plapri_r	GACTTTGGCATTAGGTGTGACATA
		Probe	Tqpro_pla	**CFR590-**TCCGGCTCACGTTATTATGGTACCG**-BHQ2**

*B. thuringiensis*	*cry1*	Forward primer	crypri_f	GCAACTATGAGTAGTGGGAGTAATTTAC
		Reverse primer	crypri_r	TTCATTGCCTGAATTGAAGACATGAG
		Probe	Tqpro_cry	**Cy5**-ACGTAAATACACT**-BHQ2-**TGATCCATTTGAAAAG**-P**

^a ^CFR590 = CALFluor Red 590, BHQ = Black Hole quencher, P = phosporylation

In order to achieve a reliable as well as rapid method for the detection of *B*. *anthracis*, *Y. pestis *and *F*. *tularensis*, the *cry1 *gene from *B. thuringiensis *was included in the multiplex qPCR assays. Inclusion of this gene permitted the development of *B*. *thuringiensis *spores as internal control for DNA extraction as well as amplification. The amount of spores that must be added to the samples before DNA extraction to obtain the desired Cq value was determined from serial dilutions of the spores.

### Specificity and coverage of strain diversity

A DNA panel from the Bacterial and Eukaryal species listed in Additional file 1 Table S1 was used to validate the specificity of the developed real-time qPCR assays. The pathogen-specific targets showed no cross-reactivity and very near relatives could be differentiated as evidenced by the absence of amplification from various members of the *Bacillus cereus *group, *Yersinia pseudotuberculosis, Y. enterocolitica *and *Francisella philomiragia*. From the latter species, one out of the four strains that were tested showed very weak amplification of the multicopy sequence IS*Ftu2*, but none of the strains showed amplification of the *F. tularensis *signature sequence *fopA*. The assays detected all available strains from the targeted organisms. Nevertheless, the genomic marker *ypo*393 was amplified from only one strain (NCTC 10329) out of four from a *Y. pestis *cluster from Nairobi. Additional information about the pathogens could be derived from the detection of particular plasmid combinations in the *B*. *anthracis *and *Y. pestis *assays, and from the detection of the *pdpD *gene [14] in the *F. tularensis *assay. This was confirmed by the anticipated absence of the *pdpD *gene in the 16 *F. tularensis holarctica *strains we tested. However, the probe designed for *pdpD *detection could not discriminate between subspecies *tularensis *and *novicida*. Based on the available sequences from *F. tularensis mediasiatica*, amplification of *pdpD *from this subspecies will occur as well, however, we did not have genomic materials to verify this. Amplification of the *pla *target from *Rattus rattus *DNA was unexpected and seemed to indicate cross-reactivity. To confirm *pla *amplification we investigated DNA from 10 rats, including 3 from the related species *Rattus norvegicus *(Additional file 1 Table S1). Sequencing of the amplification product from these samples revealed the presence of a *pla *gene highly similar to that of *Y. pestis *(99% identity), while no sequences with any homology to these sequences were encountered in the published rat genome. Therefore, the amplification does not invalidate our assay but highlights the fact that the *pla *gene alone is not a sufficient diagnostic marker for the presence of *Y. pestis*. The internal control gene *cry1 *was amplified from several *Bacillus *cultures in addition to *B. thuringiensis*.

### Efficiency, dynamic range, precision and detection limit

Ten-fold independent serial dilutions from purified target amplicons (PCR products containing target sequences) were used to generate calibration curves and calculate PCR amplification efficiencies. As shown in Table 2 efficiencies for the different targets ranged between 94.5% and 94.8% for *B. anthracis*, between 95.9% and 98.2% for *F. tularensis *and between 93.1% and 93.2% for *Y. pestis*. The efficiency for amplification of the internal control target *cry1 *varied slightly between the assays and was near that of the organism-specific targets. The reaction was linear over 6 orders of magnitude, from 1.5·10^2 ^to 1.5·10^7 ^target copies per reaction (data not shown).

**Table 2:** Precision and detection limits of the multiplex PCRs

organism	Target	Efficiency (%)	Repeatability (SD of C_q_)^a^	LOD target amplicons (copies/reaction)^b^	LOD gDNA (fg/reaction)^b^
*B*. *anthracis*	*sspE*	94.5	0.045	2.6 (1.6-7.5)	22.6 (9.9-148.5)
	
	*cya*	94.7	0.057	6.5 (3.7-19.6)	50.5 (19.1-408.3)
	
	*capB*	94.8	0.051	3.6 (2.0-10.7)	15.7 (9.9-78.9)

*F*. *tularensis*	*fopA*	98.2	0.042	7.2 (3.5-24.7)	11.8 (5.5-66.4)
	
	IS*Ftu2*	98.1	0.075	4.1 (2.2-12.8)	0.6 (0.2-3.4)
	
	*pdpD*	95.9	0.067	6.1 (3.1-20)	4.2 (2.5-25.6)

*Y. pestis*	*ypo0393*	93.1	0.057	1.7 (1.2-3.5)	116 (59.3-967.2)
	
	*caf1*	93.2	0.099	1.9 (1.3-4.1)	43.2 (23.9-277.2)
	
	*pla*	93.1	0.047	3.6 (2.2-8.9)	29.6 (13.5-191.9)

*B. thuringiensis*	*cry1*	94.6/95/92.9^c^	0.047/0.055/0.057 ^c^	ND	ND

^a ^Values represent the average from the standard deviations calculated at 5 different dilutions from 4 replicate Cqs measurements.

^b ^Values displayed represent the lowest DNA concentration at which 95% of the positive samples are detected, as calculated by using probit analysis. Shown between brackets are the 95% confidence limits of the calculated LODs.

^c ^*B. thuringiensis *internal control added to *B. anthracis*, *F. tularensis *and *Y. pestis*, respectively

ND = not determined

The precision of the different qPCR assays was calculated from 4 replicates of 5 independent dilutions. Mean Cq values and standard deviations (SD) were calculated from each dilution. As shown in Table 2 there is a high repeatability for the different targets, with SDs around 0.05 Cq. Only at very low concentrations (high Cq values) near the limit of detection, the SD exceeded 1 Cq (data not shown).

To determine the analytical sensitivity for each single target, dilutions of target amplicons near the detection limit were measured by using the developed assays. The analytical sensitivity for genomic DNA was calculated from dilutions of purified genomic DNA from selected pathogens. The fraction of positive reactions in replicate dilutions were scored and a probit analysis was used to calculate the limit of detection (LOD), which is the lowest concentration at which 95% of positive samples are detected. The LOD for single targets could be expressed as copy numbers as the target amplicons were of known size. Table 2 shows LODs of below 10 copies for the various targets. For genomic DNA, LODs based on the most sensitive target were for *B. anthracis*15.7 fg, for *F. tularensis *0.6 fg and for *Y. pestis *29.6 fg.

### Co-amplification targets in multiplex assay

**Figure 1:**
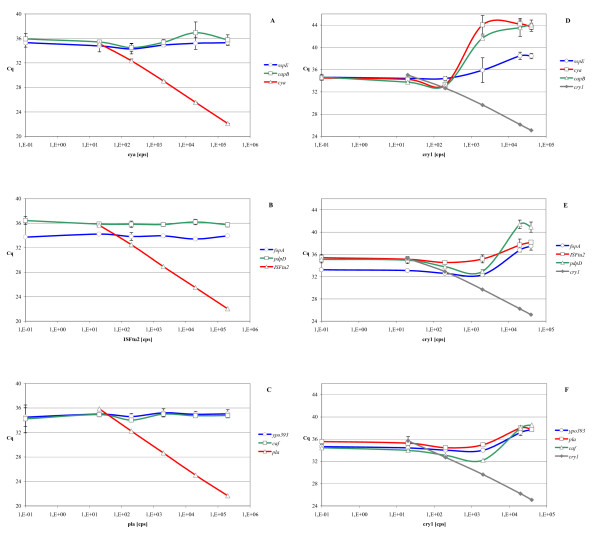
**Effect of increasing concentration differences between targets in multiplex qPCR reactions**. Dilution series of multicopy targets (A-C) or internal control target ***cry1 ***(D-F) were made in the presence of the other targets detected in each qPCR at a constant concentration near the detection limit. Triplicate multiplex qPCR measurements were performed and mean Cq values with 95% confidence limits are shown for each target.

Large concentration differences between DNA templates in a multiplex PCR may lead to competition for reaction components and impaired amplification of the rarer templates. Divergence of target concentrations could originate from different copy numbers of the targets within the pathogen genome, or from differences between the numbers of organisms that are detected simultaneously. Although there is limited copy number variation for the selected targets, multicopy sequences such as insertion sequences and plasmid genes could outnumber single-copy targets by a factor of more than 200 [3,18]. To exclude an inhibitory effect of the dominant amplification product in the multiplex reaction, dilution series of the high copy number targets (*cya*, *pla *and IS*Ftu2*) were made in the presence of a constant and low concentration of the other targets from that organism, and measured by the multiplex qPCRs (Figure 1A-C). No inhibitory effect (increasing Cq) was observed, even if the excess target considerably exceeded the maximum ratio that could be anticipated.

Significant concentration differences are possible between the pathogen specific targets and the internal control target, as these organisms could be mixed in very different quantities. Inhibition of the internal control (IC) by excess pathogen DNA is not a problem as the function of the IC is to exclude false negative results (a positive pathogen signal makes an additional IC signal irrelevant). In contrast, it is essential that inhibition of pathogen targets by the internal control is prevented. To determine the boundaries within which IC *B*. *thuringiensis *DNA could be added to pathogen DNA without interfering with the detection of low pathogen concentrations, a dilution series of the IC target amplicon (*cry1 *gene) was made in the presence of a constant and low concentration of pathogen targets and measured by the multiplex qPCRs. As shown in Figure 1D-F, the amplification of 20 copies of pathogen targets was inhibited (increasing Cq) if more than 200 copies of the internal control target were present for *B. anthracis *or more than 2000 copies for *Y. pestis *and *F. tularensis*. Moreover, rare targets were still detectable at much higher excess ratios of internal control, even though at higher Cq values.

## Discussion

### Multiplexing and the reduction of false negative and false positive results

In this report, we describe the development of multiplex qPCRs for the rapid and reliable detection of *B*. *anthracis*, *F*. *tularensis *and *Y. pestis*. The assays include a signature sequence from *B. thuringiensis *which allows the use of its spores as combined internal control for both DNA extraction and subsequent DNA amplification. As *Bacillus *spores are among the most resistant of microbial structures, DNA extraction from such spores can be considered to be a reliable indicator for successful DNA extraction from other microbes. Application of internal controls is especially important when measuring environmental samples because these tend to contain various sorts of PCR inhibitors. The internal control helps preventing false negative results, which are further reduced by the sensitivity of the methods and by the recognition of multiple signatures per organism. Multiplexing reduces the chance that the pathogen escapes detection due to modification or loss of plasmids or genes (natural or by manipulation).

Multiple diagnostic signatures per pathogen will also help reducing false positive detection, which is particularly important in complex (environmental) samples which may contain homologous genes of yet uncharacterized origin[1,2]. The genera *Bacillus*, *Francisella*, and *Yersinia *each include species ranging from nonpathogenic environmental species, through symbionts and facultative pathogens, to highly virulent human and animal pathogens. Comparative genomic sequencing and typing studies have indicated that the sequence similarity and gene composition of species having very different lifestyles can be very high [1,19-21] Also, bacterial genomes are dynamic and non-target organisms could acquire diagnostic sequences by lateral gene transfer, especially if present on plasmids [22]. An additional reason for including multiple targets is that for *B. anthracis *and *Y. pestis*, a full picture of virulence requires the detection of several markers. Although virulent *Y. pestis *usually contains three plasmids, strains deficient in one or more plasmids may cause fatal infections [6].

Assays relying on one signature sequence for the detection of a pathogen [10,23,24], suffer from the constraints mentioned above, especially when analyzing environmental samples [1]. For instance, *Y. pestis *subgroup *Pestoides *lacks the plasminogen coagulase (*pla*) gene [25] that is used as the major and sometimes only target for the detection of *Y. pestis *[23,26]. On the other hand, we found that the *pla *gene may yield false positive results in certain matrices (unpublished). In addition to relying on multiple targets, false positives are further reduced by the high specificity of the developed assays for the selected targets, which was confirmed by *in silico *and *in vitro *validations.

### Selected targets

Inclusion of chromosomal markers in addition to virulence plasmids is important due to the occurrence of *B. anthracis *and *Y. pestis strains *lacking virulence plasmids. These strains, as well as yet uncharacterized closely related environmental species, share genomic traits that could lead to misidentification. Fully virulent *B. anthracis *strains possess plasmids pXO1 and pXO2. However, the detection of plasmids only, as for instance commercial kits do, cannot detect plasmid-deficient *B. anthracis *strains such as Sterne and CDC 1014. Moreover, *B. cereus *strains carrying plasmid highly similar to those of *B. anthracis *(*B. cereus *G9241) are not correctly identified. Several chromosomal markers have been used for the detection of *B. anthracis *(e.g. *BA813*, *rpoB*, *gyrA*, *gyrB*, *saspB*, *plcR*, *BA5345*, *BA5510*), but only recently a locus was described for qPCR that did not yield any false positive results from closely related *Bacillus *[27]. We have developed an alternative chromosomal signature sequence (*sspE*) for use in real-time PCR. This marker has previously been used for specific detection of *B. anthracis*, but differentiation required melting curve analysis [8]. By selecting highly discriminating positions for primers and hydrolysis probe, we achieved specific detection without post-PCR analysis. For *Y. pestis*, it is equally important to detect chromosomal sequences in addition to its plasmids, as plasmid-deficient virulent *Y. pestis *has been described [6]. Most of the chromosomal targets that have been described previously did not differentiate *Y. pestis *from closely related *Y. pseudotuberculosis *or *Y. enterocolitica *[12]. The chromosomal signature sequence we developed for *Y. pestis *detection was based on a previous study employing comparative genome hybridization to identify chromosomal regions specific for *Y. pestis *[17]. We selected a different region than the *ypo*2088 target which was used by these authors and later by Matero et al. [16], because examination of published genomes revealed that strain *Y. pestis antiqua *(accession # CP000308) does not possess this region. Although *ypo*339 was present in all 20 *Y. pestis *sequences currently publicly available, 3 out of 4 isolates from the Nairobi cluster appeared to lack this signature sequence. Hence, although *ypo*393 is a reliable signature sequence for most *Y. pestis*, strains lacking this sequence do exist. Our results illustrate that even if signature sequences selected for diagnostic purposes are based on a considerable amount of sequences available from genomes and sequence databases, uncharacterized strain variants may exist or new variants may arise that do not posses a particular target sequence. Conversely, amplification of the *cry1 *gene from some *Bacillus *strains other than *B. thuringiensis *was not anticipated as these strains were not known to contain the plasmids carrying *cry *genes or homologues. Since it concerned related, spore-forming *Bacillus *strains, these could also be used as internal controls. The primary focus of our assays was the sensitive and specific detection of the selected pathogens, minimizing false negative and false positive results. Strain differentiation was considered to be of only secondary interest. For *F. tularensis*, sensitive detection requires detection of the multicopy sequence IS*Ftu2*. The targeted tranposase can also be present in *F. philomiragia*, but strain ATCC 225017 for instance, has only one copy with mismatches in the probe and reverse primer. This explains the very low cross-reactivity with the four strains we investigated. Nevertheless, specific detection of the species *F. tularensis *was confirmed by additional detection of the *fopA *gene [13,15]. Further subspecies information could be obtained from the *pdpD *target, which is known to be absent in subspecies holarctica (type B) [14] and was indeed not detected in the 16 strains we tested. With all targets positive, subsequent research is warranted however, as presence of this gene could also imply presence of the subspecies *novicida *and *mediasiatica *[28]. Subspecies *mediasiatica *is, similar to subspecies *holarctica*, a considerable public health threat although both species are less pathogenic compared to subspecies *tularensis*. Subspecies *novicida *is less pathogenic than the other subspecies and has been involved in only a limited number of human cases.

### Sensitivity

The analytical sensitivity for detection of the different signature sequences is very high (Table 2). Hence, the presence of only a few genomes should enable detection of the organisms of interest at 95% probability, especially when based on multicopy signature sequences. For *F. tularensis *this means that only 0.3 genomic equivalents (GE) were sufficient for the detection, considering a genome size of 1.9 megabases. For *B. anthracis *and *Y. pestis*, reliable estimates of GE could not be made due to the variable and sometimes significant contribution of plasmids to the total amount of DNA measured [3,18]. But, using approximate plasmid copy numbers, a detection limit of 4 GE for *B. anthracis *and 6 GE for *Y. pestis *can be calculated. The LODs were similar or lower than those reported previously [13,14] and lower than those of other multiplex assays for these pathogens [12]. A correlation between the copy numbers of the targeted genes and the LOD for genomic DNA can be expected. For *F. tularensis *gDNA, the LOD was indeed highest based on the detection of the single-copy *fopA *target, lower when based on the 2-copy *pdpD *and lowest when based on the approximately 20-copy IS*Ftu2 *(Table 2). Also for *Y. pestis*, an inverse correlation between gDNA LOD and expected target copy number was observed (Table 2). Nevertheless, a more pronounced difference would be expected based on the high relative abundance of *pla *carrying plasmids that has been reported [18]. Probably, the gDNA we used contained fewer plasmids, as was supported by a Cq difference between the chromosomal target and *pla *of only approximately 2 (data not shown). For *B. anthracis*, the LOD of gDNA was highest when based on the detection of the pXO1 plasmid marker *cya*, while high copy numbers for the pXO1 plasmid carrying this gene have been reported [3]. This discrepancy could be due to the gDNA preparation we used for calculating LODs. Although Coker et al. reported relative amounts of pXO1 and pXO2 of respectively 11.5 and 1.6, for the same strain we used (*B. anthracis *Vollum), variation in pXO plasmid copy numbers could also result from the growth phase at which DNA was harvested [3]. Our data correspond better to the lower plasmid copy numbers reported by other authors [29,30]. Nevertheless, all reports agree that pXO1 is present in multiple copies. The relatively high LOD for gDNA detection based on *cya *can probably partly be explained by a low amplification efficiency near the detection limit as the LOD for the detection of *cya *target amplicons is also relatively high (Table 2).

### Internal control

As was shown in Figure 1 the *cry1 *gene from *B. thuringiensis *spores can be used as internal control without affecting sensitive detection of the pathogens of interest. However, addition of more than 200 copies of *cry1 *per reaction lead to a Cq increase for the detection of the *B. anthracis *plasmid targets. For diagnostic purposes, we use a spore suspension that yields a Cq value between 32 and 35 for the detection of *cry1 *to prevent any interference with the detection of pathogen DNA. The amount of spores that needs to be added to yield this Cq should be determined for each new batch as it will vary with each new spore stock, and the DNA extraction protocol used. The observed inhibition highlights that multiplex qPCR can be problematic if it is used for the detection of mixed pathogens present in different quantities as amplification of targets from a dominant organism could inhibit the detection of an uncommon pathogen. Assays for the detection of single targets from multiple pathogens simultaneously, such as that described for *B. anthracis*, *F. tularensis *and *Y. pestis *detection [23], should therefore be carefully evaluated for this inhibition effect.

### Environmental testing

Application of the multiplex qPCR assays directly on human specimens or environmental samples could save time and prevent loss of DNA during extraction. However, we use the assays only after a DNA extraction protocol, in order to prevent unanticipated inhibition by diverse matrices. Our laboratory has compared several commercially available DNA extraction kits for use in a BSL3 facility, and selected one that combined efficient DNA extraction with ease-of-use and applicability in the restricted BSL3 environment. We have been using the developed qPCRs for the analysis of samples suspected for the presence of these pathogens with *B. thuringiensis *spores added before DNA extraction under BSL3 biosafety conditions. Hundreds of samples containing all sorts of solid materials and liquids have been analyzed without yielding false positive readings.

## Conclusion

The multiplex qPCR assays that were developed for *B. anthracis*, *F. tularensis *and *Y. pestis *allow the rapid detection of 3 pathogen-specific targets simultaneously without compromising sensitivity. Together with the application of an internal control for both DNA extraction and DNA amplification, this assures highly reliable detection, while template consumption and laboratory effort is kept at a minimum. These considerations are particularly advantageous in the context of biothreat samples which may be used for additional tests and for surge capacity during an outbreak. The detection of multiple targets decreases the chance of false-positive and false-negative results and provides additional information about virulence.

## Methods

### Selection signature sequences

An initial selection of potential signature sequences for specific detection of *B*. *anthracis*, *F*. *tularensis *and *Y. pestis *was based on previous reports and on the availability of sequences through public databases (NCBI/EMBL). The selection was based on functional and on technical criteria. Since 4 reporter dyes can be reliably differentiated by using qPCR instruments, and 1 channel was reserved for the internal control, we selected 3 signature sequences per organism. If possible, signature sequences included virulence genes since these are significant diagnostic markers. Such virulence genes are often located on plasmids. Besides plasmid-encoded targets, at least one chromosomal target was included to account for plasmid transfer and loss. Plasmids may be transferred between closely related species of *Bacillus *or *Yersinia *[8]. Plasmids can be cured from *B*. *anthracis *[31] and *Y. pestis *[6], and virulent plasmid-deficient *Y. pestis *strains occur in nature [6]. Also, near-neighbor species carrying closely-related plasmids [5] should be distinguished from *B*. *anthracis*. Finally, although *B*. *anthracis *has two plasmids that are required for virulence, there are also chromosomally encoded factors that are important for the full virulence [4]. If available, a multicopy sequence was included to enhance sensitivity. Unique targets present only in the organism of interest were preferred over targets differentiating homologues in related species only by sequence differences. Finally, an important consideration for the selection of targets was the quality of sequence information available from the public databases. This sequence quality concerned the number of sequences, their length and their coverage of strain diversity. For each potential target sequence, representative sequences were retrieved from NCBI/EMBL. BLAST searches were then performed to retrieve all homologous sequences from nucleotide and bacterial genome databases. All available sequences were aligned and consensus sequences were created using an accept level of 100% (to make sure the consensus sequence displayed all sequence variation). For *B*. *anthracis*, genes were selected on the multicopy virulence plasmids pXO1 and pXO2, and on the chromosome. The consensus alignment from the toxin gene *cya *included this gene from the homologous pBCXO1 plasmid which is present in a virulent *B*. *cereus *strain [5]. The chromosomal target for *B*. *anthracis*, the spore structural gene *sspE*, is not a unique gene as it is present in all *Bacillus*. Nevertheless, this sequence was selected since the sequence differences between *B*. *anthracis *and other species within the closely related *B*. *cereus *group were sufficient for designing highly selective oligonucleotides. Also, the presence of a substantial number of sequence entries in the databases (> 200) enabled a reliable consideration of the sequence diversity of *B*. *cereus *group isolates. For *F*. *tularensis*, the multicopy insertion sequence IS*Ftu2 *was selected for the detection of *F. tularensis*. Cross reaction with other *Francisella *species such as *F. philomiragia *could not be ruled out based on the available sequences, and a region of the outer membrane protein gene *fopA *was selected for the specific detection of all subspecies from the species *F*. *tularensis*. A specific location in the pdpD gene, which is absent from *F. tularensis *subspecies *holarctica*, was selected for the design of a probe for the detection of *F*. *tularensis *subspecies *tularensis *(type A) [14]. For *Y. pestis*, genes were selected on Y. *pestis *specific virulence plasmids pPCP1 and pMT1. The plasmid pCD1 was not used as it is shared by other pathogenic *Yersinia *species. A chromosomal sequence of unknown function that had been identified using comparative genome hybridization [17] was selected as *Y. pestis *specific chromosomal target.

Spores of *B*. *thuringiensis *were used as internal control, not only for DNA amplification but also for successful DNA extraction. This member of the *B*. *cereus *group is closely related to *B*. *anthracis *and forms similar spores, while it contains species-specific plasmids. The *B*. *thuringiensis *plasmid gene encoding insecticidal crystal proteins (*cry *genes) was used as the signature sequence for the detection of DNA released from this organism's spores.

### Sequence analysis tools, bioinformatics software

Sequences retrieved from NCBI/EMBL were organized and aligned using the software package Kodon (Applied Maths, Ghent, Belgium). Comprehensive sequence alignments were made by performing BLAST searches from the selected targets to make sure all available sequence homologues were included in the alignments. Oligonucleotides for multiplex qPCR assays and for conventional PCR assays were designed using the software package Visual Oligonucleotide Modeling Platform version 6 (DNA software Inc. Ann Arbor, USA). The design strategy for multiplex qPCR assays was as follows. First, a hydrolysis probe and primer set were designed for the *B*. *thuringiensis *internal control. Then, for each selected signature sequence a hydrolysis probe was designed, followed by the design of the corresponding primer set. A different strategy was chosen for the *B*. *anthracis *assay, because its chromosomal target *sspE *has homologues in other *Bacillus*, notably the internal control *B*. *thuringiensis*. To make sure that detection of *B*. *anthracis sspE *was highly selective, the exact positions of probe and primers were guided based on visual inspection of the alignment. Probe and primers were located in regions with mismatches between *Bacillus *species (notably between *B*. *thuringiensis *and *B*. *anthracis*), and the primers were designed such that mismatch positions were located at their highly discriminating 3'-ends. Oligonucleotides that were calculated by the design software were first checked against the consensus alignment to exclude designs not covering all sequence variants, and were then evaluated using the simulation module of Visual OMP. All oligonucleotides designed were validated *in silico *by using BLAST searches in general and microbial genomes databases (NCBI/EMBL).

### Sequencing

Sequences were obtained from the *cry1 *gene from *B. thuringiensis *strain ATCC 29730 and from the *sspE *gene from all *B. anthracis *strains in our culture collection, *B. thuringiensis *ATCC 29730 and *B. cereus *strains WSBC 10583, 10619, 10766, 10483, 10572, 10705, 10770 and 10865 (Additional file 1 Table S1). In addition, the *pla *gene was sequenced from DNA extracted from muscle tissue derived from a dissected specimen of *Rattus rattus*. Primers used for sequencing are displayed in Additional file 2 Table S2. PCR products were purified by using ExoSAP-IT (USB, Cleveland, USA) and DNA sequencing reactions were performed in both directions using BigDyeTerminator v3.1 (Applied Biosystems, Nieuwerkerk a/d IJssel, the Netherlands) on a 48-capillary 3730 DNA Analyzer sequencer (Applied Biosystems, Nieuwerkerk a/d IJssel, the Netherlands). Accession numbers: HQ222846 to HQ222861 and HQ606074.

### PCR and real-time qPCR

Oligonucleotides were synthesized by Biolegio (Biolegio, Nijmegen, the Netherlands). Conventional PCR was used to produce amplicons from signature sequences. Amplification was carried out using the HotStar Taq Master Mix Kit (Qiagen, Westburg, the Netherlands) and 400 nM primers in a total reaction volume of 50 μl. Primer sets were designed using Visual OMP software (Additional file 2 Table S2). Thermocycling conditions were as follows: 95°C for 15 min, 40 cycles at 95°C for 30 sec, 55°C for 30 sec and 72°C for 30 sec, followed by a final step at 72°C for 7 min. Thermocycling reactions were carried out in a Px2 thermal cycler (Thermo Electron Corporation, Breda, the Netherlands).

All qPCR reactions were carried out in a final volume of 20 μl containing iQ Multiplex Powermix (Bio-Rad, Veenendaal, the Netherlands), 200 nM of each primer and 100-300 nM hydrolysis probes and 3 μl of DNA template. Probes concentrations had been optimized to yield minimal spectral overlap between fluorescence level of the reporter dyes for each target in a multiplex assay and were 100, 200, 300 and 300 nM for FAM, JOE, CFR590 and Cy5 labeled probes respectively. The multiplex real-time qPCR assays had been designed for an optimal annealing temperature of 60°C and the thermal cycling conditions were as follows: First enzyme activation at 95°C for 5 min, followed by amplification and detection by 45 cycles at 95°C for 5 sec and 60°C for 35 sec. Each real-time qPCR experiment included a negative (no template) control. Measurements were carried out on a Lightcycler 480 (Roche, Almere, the Netherlands). An iQ5 (Bio-Rad) instrument was used for routine screening purposes. Analyses were performed on the instruments software: LightCycler 480 Software release 1.5.0. SP3 and iQ5 Optical Systems Software version 2.0. Cq values were calculated using the second derivative method on the LightCycler and the Base Line Subtracted Curve Fit method on the iQ5. Color compensations were carried out on both instruments as follows. PCR amplifications were performed using single primer-probe sets for each reporter dye and under identical reaction conditions as during multiplex amplification. The PCR reactions thus produced contained single dyes in relevant concentrations and these were used for color compensation runs according to the manufacturers' guidelines. Verification of PCR product sizes were carried out on the 2100 Bioanalyzer instrument (Agilent Technologies, Eindhoven, the Netherlands) using the DNA 1000 kit.

### Bacterial isolates and genomic DNA preparation

The detection limits and specificities of the assays were evaluated using genomic materials from the bacterial strains and other sources displayed in Additional file 1 Table S1. The pathogen panel included (besides a variety of Eukaryal organisms): 8 *B. anthracis *strains and 31 near relatives (22 *B. cereus*, 5 *B. thuringiensis *and 4 *B. mycoides*), 21 *F. tularensis *strains (16 subspecies *holarctica*, 4 *tularensis *and 1 *novicida*) and 4 of the closest related species *F. philomiragia*, 23 *Y. pestis *(including *Antiqua*, *Mediaevalis *and *Orientalis *biovars) and 3 strains from the closest relative *Y. pseudotuberculosis and 7 strains from Y. enterocolitica*. From most of the *B. anthracis*, *F. tularensis *and *Y. pestis *strains we only had genomic DNA (lysates) available to verify specificity of our assays. Several strains were available as live cultures in our laboratory and these were used as resource for the production of larger quantities of genomic DNA. *B. anthracis *and *Y. pestis *strains were acquired from the NCTC (National Culture Type Collection, UK) and the Pasteur Institute (France). The *Francisella holarctica *strain was a patient isolate. Other genomic materials were lysates from bacterial cultures provided by other researchers as mentioned in the acknowledgements. Cultivation of these strains was carried out in a BSL3 glove-box. Colonies from *B. anthracis*, *F. tularensis *and *Y. pestis *were grown on Columbian sheep blood agar plates and chocolate agar plates. Single colonies were transferred to liquid BHI (Brain Heart Infusion, 27 g/L) medium. After cultures had grown to visible turbidity, 1.4 ml cell culture was centrifuged and the pellet was resuspended in 250 μl TE pH 8. Cells were incubated for 30 minutes at 100°C. Lysed cultures were filtered through a 0.22 μm sterile Ultrafree-MC spinfilter (Millipore, Amsterdam, the Netherlands) and the filtrate was subsequently transported from the BSL3 facility for handling under normal laboratory conditions. Cultures from non-target bacteria that were used in the specificity panel were obtained from the culture collection at the RIVM. These cultures were cultivated under BSL2 conditions and lysates of these cultures were used for specificity testing.

DNA extraction and purification was carried out by using NucliSens Magnetic Extraction Reagents (bioMérieux, Boxtel, the Netherlands) following the manufacturers instructions. This method performed best with regard to efficiency and ease-of-use when compared to other kits. This comparison was carried out as follows. Dilution series of a mixture of genomic DNA from *B. anthracis*, *Y. pestis *and *F. tularensis*, and spores from *B. thuringiensis *were added to various powders including milk powder, soy powder, silica and maize powder, and DNA was extracted by using the powersoil DNA isolation kit (MO BIO Laboratories, Carlsbad, USA), the ultraclean microbial DNA isolation kit (MO BIO) and the NucliSens Magnetic Extraction Reagents (bioMérieux) according to the manufacturers instructions. The extracts were measured by using the developed qPCR. DNA concentrations were measured using the NanoDrop 1000 spectrophotometer (Thermo Fisher Scientific, Wilmington, USA). DNA samples were stored at 4°C for use within 1 week and at -20°C for longer storage.

### Spore suspension for use as internal control

Spore suspensions of *B. thuringiensis *strain ATCC 29730 (var. *galleriae *Heimpel) were obtained from Raven Biological Laboratories (Omaha, Nebraska, USA). These washed spores were counted by microscopy and then aliquotted and stored at 4°C. The amount of spores that needs to be added to samples to obtain suitable Cq values for this internal control must be determined empirically for each stock spore suspension. Ten-fold serial dilutions were made from the spore stock and DNA was extracted from 50 μl portions of each dilution by using the Nuclisens Magnetic Extraction Reagents (bioMérieux). The developed real-time qPCR assays were used to determine the amount of spores required for a Cq value between 32 and 35.

### Limit of detection, efficiency and repeatability

Characterization of qPCR performance was guided by the MIQE guidelines [32]. The validation was carried out by using genomic DNA as well as purified PCR amplicons including > 100 bp upstream and downstream from the qPCR amplification sites. The latter were used to compose template mixes of desired composition and quantities, while maintaining secondary structures in the primer binding regions. Detection limits (LOD) for genomic DNA were determined by using purified DNA from cultures of *B. anthracis *strain Vollum, *F. tularensis *strain *tularensis *ATCC 6223 and *Y. pestis *strain Harbin. DNA was purified from lysates of these strains. The concentration of purified genomic DNA was measured by using the NanoDrop 1000 spectrophotometer. Serial dilutions of genomic DNA were used to calculate LODs from the proportion of positive qPCRs at each dilution. Four replicates of eight serial dilutions of genomic DNA were measured by qPCR. Based on the results, an additional measurement was performed on 4 replicates of 8 novel serial dilutions. The measurements included at least one dilution with all replicates positive and one with all replicates negative. A probit analysis was performed using SPSS Statistics 18.0.0 to calculate the DNA concentration that could be measured with 95% probability.

DNA templates for measuring the detection limits from the different signature sequences were amplified from the bacterial strains mentioned above. In addition, the pdpD signature sequence from F. tularensis tularensis was amplified from ATCC 6223. To generate suitable amplicons for testing the different real-time qPCR targets, primers were designed for amplification of a signature sequence with a size of 400-800 bp, extending beyond both ends of the region amplified by the real-time qPCR. Primer sequences are displayed in Additional file 2 Table S2. After amplification, PCR products were purified and the number of DNA copies in amplicon solutions was calculated from their sizes and concentrations. Amplicon dilutions were used to calculate the LOD from the proportions of positive qPCRs at each dilution. First, 5 replicates of 8 dilutions around the estimated detection limit were measured using a mixture of equal amounts of target amplicons. Based on the results, an additional measurement was performed on 10 replicates of 8 novel dilutions. After scoring positive results, a probit analysis was performed to calculate the DNA concentration that could be measured with 95% probability.

Efficiency and repeatability were calculated from the log-linear portion of the calibration curve, covering 6 orders of magnitude. The calibration curve was made using amplicon mixtures as templates containing the signature sequences (as described before). Four replicate measurements were obtained from each dilution. For calculation of the repeatability, the lowest template concentration was not used as the standard deviation (SD) near the detection limit was not consistent with those obtained for the other concentrations.

### Dynamic range internal control

To establish a concentration range for the applicability of the internal control, serial dilutions were made of internal control *cry1 *target amplicon (0, 2·10^1^, 2·10^2^, 2·10^3^, 2·10^4^, 4·10^4 ^copies per reaction) in the presence of a mixture of the 3 organism specific target amplicons, each at a concentration of 20 copies per reaction. These target amplicon mixtures were amplified in triplicate by using the developed qPCR assays and Cq values were used to infer possible inhibition of PCR amplification. To investigate inhibitory effects on the amplification of organism-specific targets, triplicate measurements were performed on amplicons of the multicopy targets (*cya*, *pla *and IS*Ftu2*) diluted as above in the presence of the 2 other organism-specific target amplicons, each at a concentration of 20 copies per reaction.

## Authors' contributions

IJ: conceived the study and designed the experiments, performed oligonucleotide designs and statistical analyses, interpreted experimental results and wrote the manuscript; RAH: participated in the design of the experiments, carried out and interpreted the experimental work, and helped to draft the manuscript; JMB: helped carrying out experiments; BvR: coordinated the work. All authors read and approved the final manuscript.

## Supplementary Material

Additional file 1 **Table S1 - Panel of organisms used for coverage and specificity analysis**. This table lists the different strains of the targeted pathogens, their close relatives, and a selection of other Bacteria and Eukarya that were used to validate the specificity of the developed multiplex qPCR assays. Amplification results are given for each signature sequence. Click here for file

Additional file 2 **Table S2 - Primer sequences for conventional PCR**. This table displays the primers that were developed for convential PCR. These primers were applied for sequencing and for the production of target amplicons that were used for assay validation. Click here for file
