# Supplementary material for: Reliable detection of Bacillus anthracis, Francisella tularensis and Yersinia pestis by using multiplex qPCR including internal controls for nucleic acid extraction and amplification
Source: BMC Microbiol. 2010 Dec 8;10:314. doi: 10.1186/1471-2180-10-314 (PMC3016324; doi:10.1186/1471-2180-10-314)
Supplement: Additional file 1 — Table S1 - Panel of organisms used for coverage and specificity analysis. This table lists the different strains of the targeted pathogens, their close relatives, and a selection of other Bacteria and Eukarya that were used to validate the specificity of the developed multiplex qPCR assays. Amplification results are given for each signature sequence. [file 1471-2180-10-314-S1.DOC]

## Additional file 1

**Table S1 - Panel of organisms used for coverage and** specificity analysis

| **Species** | **Strain** | **Strain details^a^** | **Multiplex PCR results^b^** | | | | | | | | | |
| --- | --- | --- | --- | --- | --- | --- | --- | --- | --- | --- | --- | --- |
|  |  |  | *sspE* | *cya* | *capB* | *fopA* | IS*Ftu2* | *pdpD* | *ypo393* | *caf1* | *pla* | *Cry1* |
| *Bacillus anthracis* | NCTC 109 | Shaving brush, London, 1920 | + | - | - | - | - | - | - | - | - | - |
|  | NCTC 8234 | Weybridge, 1951 (Sterne) | + | + | - | - | - | - | - | - | - | - |
|  | NCTC 7753 | Bradford, 1948 | + | - | + | - | - | - | - | - | - | - |
|  | NCTC 7752 | Bradford, 1948 | + | - | + | - | - | - | - | - | - | - |
|  | NCTC 5444 | St Marys Hospital, London, 1938 | + | - | + | - | - | - | - | - | - | - |
|  | NCTC 2620 | Chinese hide, London, 1928 | + | + | + | - | - | - | - | - | - | - |
|  | NCTC 1328 | London, 1922 | + | + | - | - | - | - | - | - | - | - |
|  | NCTC 10340 | Cow, Edinburgh, 1963 (Vollum) | + | + | + | - | - | - | - | - | - | - |
| *Francisella* | ATCC 6223 | Human lymph node, Utah USA, 1920 | - | - | - | + | + | + | - | - | - | - |
| *tularensis* | HFSC4 | Dugway, Utah, USA | - | - | - | + | + | + | - | - | - | - |
| *subsp. tularensis (A)* | HFSC15 (SR/1) |  | - | - | - | + | + | + | - | - | - | - |
|  | SEV23 | Ixodes tick, Australia, 1990 | - | - | - | + | + | + | - | - | - | - |
| *Francisella* | BD07-537 | Clinical isolate, Netherlands, 2007 | - | - | - | + | + | - | - | - | - | - |
| *tularensis* | FT7 | Clinical isolate, Spain, 1998 | - | - | - | + | + | - | - | - | - | - |
| *subsp. holarctica (B)* | FT8 | Clinical isolate, Spain, 1998 | - | - | - | + | + | - | - | - | - | - |
|  | FT9 | Clinical isolate, Spain, 1998 | - | - | - | + | + | - | - | - | - | - |
|  | FT10 | Clinical isolate, Spain, 1998 | - | - | - | + | + | - | - | - | - | - |
|  | LVS | Live vaccine strain, Russia | - | - | - | + | + | - | - | - | - | - |
|  | T8 | Tick, Japan, 1957 | - | - | - | + | + | - | - | - | - | - |
|  | T30 | European brown hare, Sweden, 2005 | - | - | - | + | + | - | - | - | - | - |
|  | T21 (FSC089) | Human blood, Norway, 1989 | - | - | - | + | + | - | - | - | - | - |
|  | T20 | Clinical isolate, France, 1994 | - | - | - | + | + | - | - | - | - | - |
|  | T32 | European brown hare, Sweden, 2005 | - | - | - | + | + | - | - | - | - | - |
|  | T34 | Hare, Sweden, 2000 | - | - | - | + | + | - | - | - | - | - |
|  | T4 | Vaccin strain SBL vaccin | - | - | - | + | + | - | - | - | - | - |
|  | T23 | Clinical isolate, Sweden, 1998 | - | - | - | + | + | - | - | - | - | - |
|  | T28 | Hare, Sweden, 2003 | - | - | - | + | + | - | - | - | - | - |
|  | T11 | Clinical isolate, Sweden, 1984 | - | - | - | + | + | - | - | - | - | - |
| *Francisella tularensis*  *subsp. novicida* | ATCC 15482 | Water, USA, 1951 | - | - | - | + | + | + | - | - | - | - |
|  |  |  |  |  |  |  |  |  |  |  |  |
|  |  |  |  |  |  |  |  |  |  |  |  |
| *Yersinia pestis* | Kenya 164 | Biovar antiqua, Kenya, <1952 | - | - | - | - | - | - | + | + | + | - |
|  | Harbin | Biovar mediaevalis, China, <1948 | - | - | - | - | - | - | + | + | + | - |
|  | KIM3 | Biovar mediaevalis, Kurdistan, 1968 | - | - | - | - | - | - | + | + | - | - |
|  | Madagascar 34-94 | Biovar orientalis, Madagascar | - | - | - | - | - | - | + | + | + | - |
|  | Madagascar 73-93 | Biovar orientalis, Madagascar | - | - | - | - | - | - | + | + | + | - |
|  | Nha Trang 63-115 | Biovar orientalis, Vietnam | - | - | - | - | - | - | + | - | + | - |
|  | Hamburg 19 | Biovar orientalis, Hamburg | - | - | - | - | - | - | + | + | - | - |
|  | EV 76 | Biovar orientalis, Madagascar | - | - | - | - | - | - | + | + | + | - |
|  | CCUG 32133 | Biovar orientalis, Madagascar | - | - | - | - | - | - | + | + | + | - |
|  | Peru 184 | Peru | - | - | - | - | - | - | + | + | + | - |
|  | Tjiwidej | Indonesia | - | - | - | - | - | - | + | + | - | - |
|  | NCTC 2028 | Java, 1925 | - | - | - | - | - | - | + | + | + | - |
|  | NCTC 5923 | Java, 1939 | - | - | - | - | - | - | + | + | + | - |
|  | NCTC 144 | Innoculated guinea pig, London, 1920 | - | - | - | - | - | - | + | + | + | - |
|  | NCTC 570 | Human case of bubonic plague, Bombay, 1920 | - | - | - | - | - | - | + | + | + | - |
|  |  |  |  |  |  |  |  |  |  |  |  |
|  | NCTC 2868 | Blood culture, Bombay, 1928 | - | - | - | - | - | - | + | + | + | - |
|  | NCTC 5924 | Java, 1939 | - | - | - | - | - | - | + | + | + | - |
|  | NCTC 8775 | Manchester, 1953 | - | - | - | - | - | - | + | + | + | - |
|  | NCTC 8779 | Manchester, 1953 | - | - | - | - | - | - | + | + | + | - |
|  | NCTC 10029 | Human bubonic plague, Nairobi, 1958 | - | - | - | - | - | - | - | + | + | - |
|  | NCTC 10030 | Human fatal bubonic plague, Nairobi, 1958 | - | - | - | - | - | - | - | + | + | - |
|  | NCTC 10329 | Nairobi, 1963 | - | - | - | - | - | - | + | + | + | - |
|  | NCTC 10330 | Nairobi, 1963 | - | - | - | - | - | - | - | + | + | - |
| *Bacillus atrophaeus* | ATCC 9372 |  | - | - | - | - | - | - | - | - | - | - |
| *Bacillus cereus* | ATCC 11778 | NCIB Aberdeen, 1962 | - | - | - | - | - | - | - | - | - | - |
|  | ATCC 10702 |  | - | - | - | - | - | - | - | - | - | - |
|  | NCTC 2599 |  | - | - | - | - | - | - | - | - | - | - |
|  | BD00-252 | Clinical isolate, Netherlands, 2000 | - | - | - | - | - | - | - | - | - | - |
|  | BD02-550 | Clinical isolate, Netherlands, 2002 | - | - | - | - | - | - | - | - | - | - |
|  | BD03-229 | Clinical isolate, Netherlands, 2003 | - | - | - | - | - | - | - | - | - | - |
|  | BD05-273 | Clinical isolate, Netherlands, 2005 | - | - | - | - | - | - | - | - | - | (+) |
|  | WSBC 10530 | Vomit from cooked rice, USA or UK, 1972 | - | - | - | - | - | - | - | - | - | (+) |
|  | WSBC 10536 | Indian rice dish, Germany/Pasau, 2001 | - | - | - | - | - | - | - | - | - | (+) |
|  | WSBC 10583 | Cooked rice, 1998 | - | - | - | - | - | - | - | - | - | (+) |
|  | WSBC 10619 | Dialysis liquid, Finland, 1998 | - | - | - | - | - | - | - | - | - | (+) |
|  | WSBC 10766 | Mixed spices, Norway, 1999 | - | - | - | - | - | - | - | - | - | (+) |
|  | WSBC 10286 | Cream, 1994 | - | - | - | - | - | - | - | - | - | (+) |
|  | WSBC 10483 | Tobacco, India, 1997 | - | - | - | - | - | - | - | - | - | (+) |
|  | WSBC 10566 | Foodborne outbreak, UK, 1972 | - | - | - | - | - | - | - | - | - | - |
|  | WSBC 10572 | Raw milk, Sweden, 1998 | - | - | - | - | - | - | - | - | - | (+) |
|  | WSBC 10705 | Baby food, Germany, 1993 | - | - | - | - | - | - | - | - | - | - |
|  | WSBC 10763 | Topping on steak, Norway, 1998 | - | - | - | - | - | - | - | - | - | (+) |
|  | WSBC 10770 | Ice-cream, Norway, 1999 | - | - | - | - | - | - | - | - | - | - |
|  | WSBC 10865 | Spoiled cheese, Canada, 1930 | - | - | - | - | - | - | - | - | - | - |
|  | ATCC 10876 | Contaminated bottle | - | - | - | - | - | - | - | - | - | - |
|  | ATCC 7064 | Blood | - | - | - | - | - | - | - | - | - | (+) |
| *Bacillus coagulans* |  | Purchased at Raven Labs, USA | - | - | - | - | - | - | - | - | - | + |
| *Bacillus* | ATCC 9945 | Flour, USA | - | - | - | - | - | - | - | - | - | - |
| *licheniformis* |  |  |  |  |  |  |  |  |  |  |  |  |
| *Bacillus megaterium* | ATCC 8245 |  | - | - | - | - | - | - | - | - | - | (+) |
|  | ATCC 14581 | Edinburgh, 1963 | - | - | - | - | - | - | - | - | - | - |
| *Bacillus mycoides* | ATCC 6462 | Soil | - | - | - | - | - | - | - | - | - | + |
|  | BD03-135 | Clinical isolate, 2003 | - | - | - | - | - | - | - | - | - | - |
|  | BD03-238 | Clinical isolate, 2003 | - | - | - | - | - | - | - | - | - | - |
|  | BD05-407 | Clinical isolate, 2005 | - | - | - | - | - | - | - | - | - | (+) |
| *Bacillus pumilus* | ATCC 27142 |  | - | - | - | - | - | - | - | - | - | - |
| *Bacillus subtilis* | ATCC 6633 |  | - | - | - | - | - | - | - | - | - | - |
| *Bacillus thuringiensis* | ATCC 29730 | var. galleriae Heimpel | - | - | - | - | - | - | - | - | - | + |
|  | ATCC 10792 | var. berliner, Mediterranean flour moth | - | - | - | - | - | - | - | - | - | - |
|  | BD07-271 | var. kurstaki | - | - | - | - | - | - | - | - | - | - |
|  | BD07-272 | var. aizawai | - | - | - | - | - | - | - | - | - | + |
|  | BD07-273 | var. galleriae | - | - | - | - | - | - | - | - | - | + |
| *Enterobacter cloacae* | NCTC 13168 |  | - | - | - | - | - | - | - | - | - | - |
| *Escherichia coli* | ATCC 25922 | Clinical isolate, 1946 | - | - | - | - | - | - | - | - | - | - |
| *Francisella* | CCUG 13404 | Clinical isolate, Switzerland, 1979 | - | - | - | - | (+) | - | - | - | - | - |
| *philomiragia* | CCUG 19701 | River, Utah USA | - | - | - | - | - | - | - | - | - | - |
|  | CCUG 12603 | Human abscess, Sweden, 1982 | - | - | - | - | - | - | - | - | - | - |
|  | ATCC 25015 | Moribund muskrat, Utah USA | - | - | - | - | - | - | - | - | - | - |
| *Pseudomonas* | ATCC 15442 |  | - | - | - | - | - | - | - | - | - | - |
| *Aeruginosa* | ATCC 27853 | Blood culture, 1969 | - | - | - | - | - | - | - | - | - | - |
| *Salmonella enterica* | ATCC 14028 | Serovar Typhimurium. Bovine septicaemic | - | - | - | - | - | - | - | - | - | - |
| *subsp. enterica* |  | liver, 1987 |  |  |  |  |  |  |  |  |  |  |
|  | ATCC 13076 | Serovar Enteritidis | - | - | - | - | - | - | - | - | - | - |
| *Pantoea* | BD94-368 | Clinical isolate, 1994 | - | - | - | - | - | - | - | - | - | - |
| *agglomerans* | BD94-360 | Clinical isolate, 1994 | - | - | - | - | - | - | - | - | - | - |
|  | BD91-940 | Clinical isolate, 1991 | - | - | - | - | - | - | - | - | - | - |
| *Yersinia* | BD94-26 | Clinical isolate, 1994 | - | - | - | - | - | - | - | - | - | - |
| *enterocolitica* | BD93-563 | Clinical isolate, 1993 | - | - | - | - | - | - | - | - | - | - |
|  | BD94-179 | Clinical isolate, 1994 | - | - | - | - | - | - | - | - | - | - |
|  | BD94-186 | Clinical isolate, 1994 | - | - | - | - | - | - | - | - | - | - |
|  | BD94-321 | Clinical isolate, 1994 | - | - | - | - | - | - | - | - | - | - |
|  | BD93-645 | Clinical isolate, 1993 | - | - | - | - | - | - | - | - | - | - |
|  | ATCC 9610 | Human tissue, France, 1932 | - | - | - | - | - | - | - | - | - | - |
| *Yersinia frederiksenii* | BD89-1545 | Clinical isolate, 1998 | - | - | - | - | - | - | - | - | - | - |
| *Yersinia* | BD90-58 | Clinical isolate, 1990 | - | - | - | - | - | - | - | - | - | - |
| *pseudotuberculosis* | BD90-89 | Clinical isolate, 1990 | - | - | - | - | - | - | - | - | - | - |
|  | BD90-101 | Clinical isolate, 1990 | - | - | - | - | - | - | - | - | - | - |
| *Bos taurus* | 0469 | Tissue experimental animal, Netherlands, | - | - | - | - | - | - | - | - | - | - |
|  |  | 2009 |  |  |  |  |  |  |  |  |  |  |
| *Chrysops relictus* | I | Tissue, Netherlands, 2009 | - | - | - | - | - | - | - | - | - | - |
|  | II | Tissue, Netherlands, 2009 | - | - | - | - | - | - | - | - | - | - |
| *Homo sapiens* | Volunteer 8 | Blood, Netherlands, 2009 | - | - | - | - | - | - | - | - | - | - |
|  | Volunteer 10 | Blood, Netherlands, 2009 | - | - | - | - | - | - | - | - | - | - |
|  | Volunteer 11 | Blood, Netherlands, 2009 | - | - | - | - | - | - | - | - | - | - |
|  | Volunteer 12 | Blood, Netherlands, 2009 | - | - | - | - | - | - | - | - | - | - |
| *Ixodes ricinus* |  | Collected from human host, Netherlands, 2009 | - | - | - | - | - | - | - | - | - | - |
|  |  |  |  |  |  |  |  |  |  |  |  |
| *Klebsiella* | BD05-258 | Clinical isolate, 2005 | - | - | - | - | - | - | - | - | - | - |
| *pneumoniae* |  |  |  |  |  |  |  |  |  |  |  |  |
| *Mus musculus* |  | Tissue, Netherlands, 2009 | - | - | - | - | - | - | - | - | - | - |
| *Ovis aries* | Twello 67 | Tissue slaughterhouse, Netherlands, 2009 | - | - | - | - | - | - | - | - | - | - |
| *Rattus norvegicus* | 08603 | Tissue, Netherlands, 2009 | - | - | - | - | - | - | - | - | - | - |
|  | 08604 | Tissue, Netherlands, 2009 | - | - | - | - | - | - | - | - | - | - |
|  | 08605 | Tissue, Netherlands, 2009 | - | - | - | - | - | - | - | - | - | - |
| *Rattus rattus* | 08401 | Tissue, Netherlands, 2009 | - | - | - | - | - | - | - | - | (+) | - |
|  | 08402 | Tissue, Netherlands, 2009 | - | - | - | - | - | - | - | - | (+) | - |
|  | 08403 | Tissue, Netherlands, 2009 | - | - | - | - | - | - | - | - | (+) | - |
|  | 08404 | Tissue, Netherlands, 2009 | - | - | - | - | - | - | - | - | (+) | - |
|  | 08405 | Tissue, Netherlands, 2009 | - | - | - | - | - | - | - | - | - | - |
|  | 08406 | Tissue, Netherlands, 2009 | - | - | - | - | - | - | - | - | - | - |
|  | 08407 | Tissue, Netherlands, 2009 | - | - | - | - | - | - | - | - | (+) | - |
| *Sus scrofa* | 566 | Tissue slaughterhouse, Netherlands, 2009 | - | - | - | - | - | - | - | - | - | - |

^a^ The names of the countries or towns are those used at the time of the strain isolation and have been kept for strain designation.

^b^ Good amplification (Cq < 25), **+** ; weak amplification (Cq > 33), (**+**); no amplification, **-**
