# Supplementary material for: Reliable detection of Bacillus anthracis, Francisella tularensis and Yersinia pestis by using multiplex qPCR including internal controls for nucleic acid extraction and amplification
Source: BMC Microbiol. 2010 Dec 8;10:314. doi: 10.1186/1471-2180-10-314 (PMC3016324; doi:10.1186/1471-2180-10-314)
Supplement: Additional file 2 — Table S2 - Primer sequences for conventional PCR. This table displays the primers that were developed for convential PCR. These primers were applied for sequencing and for the production of target amplicons that were used for assay validation. [file 1471-2180-10-314-S2.DOC]

## Additional file 2

**Table S2 - Primer sequences for conventional PCR**

| *Application* | *Target* | *Oligo name* | *Sequence 5’-3’* |
| --- | --- | --- | --- |
| Sequencing | *sspE* | spE_f | gaattggttacattattgattgtgga |
|  |  | spE_r | gagaagtgtttttctttcattac |
|  | *cry1* | Btc1ba_f | aaaatctactaatcttggctctgg |
|  |  | Btc1ba_r | attctctttgaatacgtcatcgcc |
|  |  | Btc1bb_f | tagatcgaattgaatttgttccgg |
|  |  | Btc1bb_r | cagattcttttgcctctttataaa |
|  |  | Btc1bc_f | ggatgtacagacttaaatgaggac |
|  |  | Btc1bc_r | tatcctcgattacgagaagtgtac |
| Generating | *sspE* | spE_f | TGAATTGGTTACATTATTGATTGTGGA |
| target |  | spE_r | GAGAAGTGTTTTTCTTTCATTAC |
| amplicons | *capB* | caBtrg_f | CGCGAATGATATATTGGTTTACTGACGAG |
|  |  | caBtrg_r | TCCTCATCAATCCCAAGAGCCTCT |
|  | *cya* | cyatrg_f | AGGAAACCGGATTCAACTAAGGG |
|  |  | cyatrg_r | TGGATTCCACGAAATATTGATATTTTACG |
|  | *fopA* | foAtrg_r | GATGAGATTAAAAAGTATTGTTATAGCTAC |
|  |  | foAtrg_f | CTGCAGCATATGGAGTAAACATAGTAT |
|  | IS*Ftu2* | isftrg_f | ATGGTAAATATAGATCAATACATAAGCGTT |
|  |  | isftrg_r | GGTTTTATCAAATCTAGAGAATACTCTTC |
|  | *pdpD* | pdDtrg_f | CTGATGACTTAATTGGTTTTTCAGATGCTA |
|  |  | pdDtrg_r | GTTTGTGAGTAGTTAATTAAAGTATCTTGAAC |
|  | *ypo0393* | ypo93trg_f | CTAGCATAAGAAACAAGCCTGCATCAA |
|  |  | ypo93trg_r | CCTTATTAGTTTCGGGTGTGAAAATTTTAG |
|  | *caf1* | caftrg_f | TGCATTATTTGGAACTATTGCAACTGCTA |
|  |  | caftrg_r | CCGCCTTTGGAACCAATTGAGC |
|  | *pla* | platrg_f | ATGAAAATCAATCTGAGTGGACAGATCAC |
|  |  | platrg_r | CAGAAGCGATATTGCAGAC |
|  | *cry1* | BtRcry_f | aaaatctactaatcttggctctgg |
|  |  | BtRcry_r | attctctttgaatacgtcatcgcc |
